# Combining abbreviated literature searches with single-reviewer screening: three case studies of rapid reviews

**DOI:** 10.1186/s13643-020-01413-7

**Published:** 2020-07-18

**Authors:** Lisa Affengruber, Gernot Wagner, Siw Waffenschmidt, Stefan K. Lhachimi, Barbara Nussbaumer-Streit, Kylie Thaler, Ursula Griebler, Irma Klerings, Gerald Gartlehner

**Affiliations:** 1 Department for Evidence-based Medicine and Evaluation, Cochrane Austria, Danube University Krems, Dr. Karl Dorrek Strasse 30, 3500 Krems, Austria; 2 Department of Family Medicine, Care and Public Health Research Institute (CAPHRI), Maastricht University, Peter Debyeplein 1, 6229 HA Maastricht, The Netherlands; 3 Information Management Unit, Institute for Quality and Efficiency in Health Care (IQWiG), Im Mediapark 8, 50670 Cologne, Germany; 4 Research Group Evidence-Based Public Health, Leibniz Institute for Epidemiology and Prevention Research (BIPS), Bremen, Germany; 5 Health Sciences Bremen, Institute for Public Health and Nursing, University of Bremen, Achterstraße 30, 28359 Bremen, Germany; 6 Medical Department I, Hanusch Krankenhaus der Wiener Gebietskrankenkasse, Heinrich-Collin-Straße 30, 1140 Vienna, Austria; 7 RTI International, 3040 Cornwallis Road, PO Box 12194, Research Triangle Park, North Carolina 27709-2194 USA

**Keywords:** Systematic review, Rapid review, Evidence synthesis, Health care decision-making

## Abstract

**Background:**

Decision-makers increasingly request rapid answers to clinical or public health questions. To save time, personnel, and financial resources, rapid reviews streamline the methodological steps of the systematic review process. We aimed to explore the validity of a rapid review approach that combines a substantially abbreviated literature search with a single-reviewer screening of abstracts and full texts using three case studies.

**Methods:**

We used a convenience sample of three ongoing Cochrane reviews as reference standards. Two reviews addressed oncological topics and one addressed a public health topic. For each of the three topics, three reviewers screened the literature independently. Our primary outcome was the change in conclusions between the rapid reviews and the respective Cochrane reviews. In case the rapid approach missed studies, we recalculated the meta-analyses for the main outcomes and asked Cochrane review authors if the new body of evidence would change their original conclusion compared with the reference standards. Additionally, we assessed the sensitivity of the rapid review approach compared with the results of the original Cochrane reviews.

**Results:**

For the two oncological topics (case studies 1 and 2), the three rapid reviews each yielded the same conclusions as the Cochrane reviews. However, the authors would have had less certainty about their conclusion in case study 2. For case study 3, the public health topic, only one of the three rapid reviews led to the same conclusion as the Cochrane review. The other two rapid reviews provided insufficient information for the authors to draw conclusions. Using the rapid review approach, the sensitivity was 100% (3 of 3) for case study 1. For case study 2, the three rapid reviews identified 40% (4 of 10), 50% (5 of 10), and 60% (6 of 10) of the included studies, respectively; for case study 3, the respective numbers were 38% (8 of 21), 43% (9 of 21), and 48% (10 of 21).

**Conclusions:**

Within the limitations of these case studies, a rapid review approach that combines abbreviated literature searches with single-reviewer screening may be feasible for focused clinical questions. For complex public health topics, sensitivity seems to be insufficient.

## Background

Rapid reviews streamline the methodological steps of the systematic review process to provide quicker answers to decision-makers’ relevant questions and save personnel and financial resources. Although the reliability of rapid review findings might be limited compared to that of systematic reviews, decisions-makers increasingly request rapid review products to answer urgent clinical or public health questions [1, 2]. For example, Australian policy agencies used 134 of 150 commissioned rapid reviews (89%) to decide the details of a policy or program, identify priorities for future action, or communicate evidence to stakeholders [3]. By using rapid reviews, decision-makers consciously accept a higher degree of uncertainty of results in exchange for an accelerated evidence synthesis product [4].

To date, agreements about minimum methodological criteria and clear definitions for rapid reviews are still lacking [5, 6], and the methodological quality of rapid reviews varies [7]. Previous studies indicate highly heterogeneous approaches when it comes to methodological shortcuts of rapid reviews [8, 9]. Two stages of the systematic review process that are often subject to methodological shortcuts in rapid reviews are literature searches and the screening of abstracts and full texts [9]. These two steps are labor-intensive and closely related. Method studies assessing the impact of specific methodological shortcuts during literature searches and screening on the validity of results and conclusions are, however, still rare. A recent study by Nussbaumer-Streit et al. [10] indicated that, in most instances, searches of at least two electronic databases or a combination of a single database with the search of reference lists lead to the same direction of conclusions as comprehensive literature searches. Waffenschmidt et al. [11] proposed an even more abbreviated electronic literature search approach than Nussbaumer-Streit et al. [10]. A simple-structured Boolean search combined with the “similar articles” function in PubMed was a valid and reliable technique to identify published randomized controlled trials (RCTs) [11]. The authors proposed that their approach could be used as an add-on to preliminary searches for the validation of search strategies or as a routine component in any systematic search [11].

Few studies have investigated the impact of single-reviewer literature screening [12–15]. A systematic review found that a median of 5% of the relevant studies was missed by single-reviewer screening (range 0 to 58%) [15]. None of the available studies, however, assessed the impact of “missed studies” on the results and conclusions of the evidence syntheses. To date, the cumulative effects of abbreviated literature searches and single-reviewer screening have not been tested.

The objective of our study was to explore the validity of a rapid review approach that combines a substantially abbreviated literature search with a single-reviewer screening of the identified records. We assessed this rapid review strategy for two focused oncological topics and a population-based public health topic.

## Methods

To address our objectives, we used a diagnostic test accuracy framework that assessed the performance of a rapid review approach to identify the included studies of three Cochrane reviews.

### Reference standards

We used a convenience sample of three ongoing Cochrane reviews [16–18] (see Table 1) as reference standards to explore the validity of a rapid review approach. We decided to use Cochrane reviews as comparators because they adhere to a rigorous systematic review of methodological standards [19]. Two of these reviews addressed the pharmacological treatments of malignant diseases (nivolumab for Hodgkin’s lymphoma [16] and early versus deferred androgen suppression for advanced prostate cancer [18]); the third review was on a public health policy topic (unconditional cash transfers for reducing poverty and vulnerabilities [17]). We used ongoing reviews so that investigators conducting the rapid reviews were masked regarding the final inclusions of the Cochrane reviews.

**Table 1:** Overview of the Cochrane reviews used as reference standards

Case study	Author, year of publication	Cochrane review title Cochrane review group	Aim	Number of studies (number of publications) included in the Cochrane review
1	Goldkuhle et al. 2018 [16]	Nivolumab for adults with Hodgkin’s lymphoma Hematological malignancies group	To assess the benefits and harms of nivolumab in adult individuals with Hodgkin’s lymphoma	3 (26)
2	Kunath et al. 2019 [18]	Early versus deferred androgen suppression for treating advanced prostate cancer Urology group	To assess the effects of early versus deferred androgen suppression therapy for advanced hormone-sensitive prostate cancer	10 (53)
3	Pega et al. 2017 [17]	Unconditional cash transfers for reducing poverty and vulnerabilities Public health group	To assess the effect of unconditional cash transfers for reducing poverty and vulnerabilities on the use of health services and health outcomes in children and adults in low- and middle-income countries	21 (68)

### General methodological approach

**Fig. 1:**
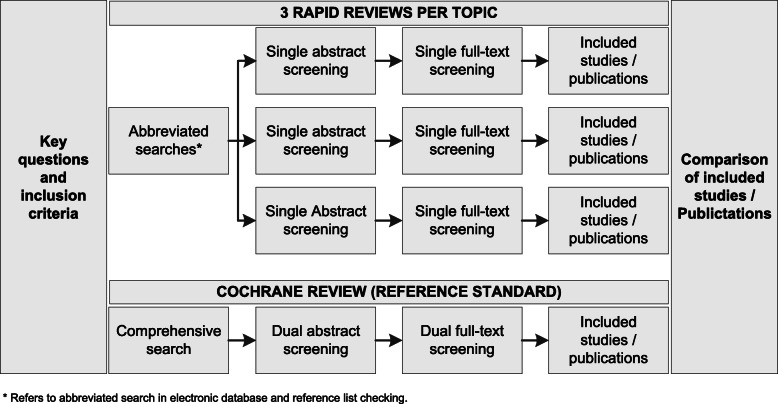
Flow chart of the rapid review literature search and identification process

For each topic, we conducted three independent rapid reviews (nine in total) to mitigate the investigators’ subjective decisions during study screening. Literature searches in electronic databases were the same for each rapid review, but three different investigators independently screened the literature and scanned the reference lists for relevant studies. For each rapid review, we used the same key questions and eligibility criteria as the Cochrane reviews [16–18]. We randomly assigned the topic order to rapid reviewers to mitigate learning effects. The deliverable for each rapid review was a table with the included studies without data abstraction, risk of bias assessment, or synthesis of the evidence. Figure 1 illustrates the rapid review approach in more detail.

### Abbreviated literature searches

Instead of comprehensive systematic literature searches in multiple databases, we employed an abbreviated electronic search approach, as proposed by Waffenschmidt et al. [11]. The first part of this search technique involves a simple-structured Boolean search in PubMed. Our search terms referred to population and the intervention of the original research questions in the Cochrane reviews’ protocols [20–22]. Terms for the populations and interventions were linked with “AND” and combined with PubMed’s Clinical Query feature (RCT filter: category: therapy, scope: narrow) [11]. If nonrandomized studies were eligible for inclusion, we applied suitable high-specificity study filters.

The second part of the abbreviated electronic search strategy used the “similar articles” function in PubMed. The starting point for this PubMed function was a “starter set” of highly relevant, published studies. We asked the authors of the respective Cochrane reviews to provide us with at least three such publications. These publications had to be primary studies cited in the review protocol, meaning the systematic review team was aware of them before they began their own formal literature searches. We applied the “similar articles” function in PubMed for each of these publications. We retrieved the first 20 publications of each “similar articles” search (which always includes the original publication as well). An experienced information specialist conducted all searches (SW). Additional file 1 provides the detailed search strategies.

Further, each rapid reviewer manually checked the reference lists of included articles for potentially relevant additional publications. Reviewers retrieved full texts of potentially relevant publications that had not been detected by electronic literature searches.

### Literature screening

We allocated three reviewers for each topic. Each reviewer independently screened the retrieved abstracts and full texts. The reviewers applied the same inclusion and exclusion criteria as the respective Cochrane review teams [16–18]. We used the web-based software tool Covidence (www.covidence.org, Veritas Health Innovation) for the abstract and full-text screening.

### Outcomes

The primary outcome of this study was the proportion of concordant conclusions between the rapid reviews and the Cochrane reviews. To address this outcome, we applied a method by Nussbaumer-Streit et al. [23]. We considered studies as identified if at least one publication of a relevant study had been included. Based on the identified studies, we recalculated the meta-analyses of all the outcomes reported in the original Cochrane review’s main summary of findings table. We created a new summary of finding tables by updating the number of studies identified through our abbreviated rapid review approach and the new effect estimates. We did not, however, change Cochrane authors’ certainty of evidence ratings. In the revised summary of findings tables, we highlighted changes compared with the original Cochrane summary of findings tables. We set up an online questionnaire (www.surveymonkey.com) for the Cochrane authors, to present them the new summary of findings tables and asked them to determine whether the evidence identified with the rapid reviews would lead to a different or the same conclusion. The authors could select one of the following options in the online questionnaire: (a) The conclusion does not change, (b) I would draw the same conclusion but with less certainty, (c) I would draw a different conclusion (in the opposite direction) with high certainty, (d) I would draw a different conclusion (in the opposite direction) but with little certainty, or (e) I can no longer draw a conclusion.

Secondary outcomes were the sensitivity of the abbreviated electronic searches as well as the sensitivity of the entire rapid review approach (the combination of the abbreviated electronic searches, reference list checking, and single-reviewer literature screening) to detect studies included in the reference standard Cochrane reviews. Other secondary outcomes were the number of abstracts screened in the rapid reviews compared to the Cochrane reviews and the time that reviewers needed for literature screening and checking reference lists.

### Data management and analysis

We imported all the records into a bibliographic database (EndNote X8, Clarivate Analytics, USA). We uploaded the citations and full-text articles into Covidence and tracked the study selection process online. To compare the included publications of the Cochrane reviews and rapid reviews, we used Microsoft Excel (2016) spreadsheets.

We performed descriptive statistics to present findings of dichotomous (proportions) and continuous (median and interquartile ranges) outcomes. We calculated the sensitivities with 95% confidence intervals for the proportion of correctly identified studies for the abbreviated electronic searches alone as well as for the combination of an abbreviated literature search with a single-reviewer literature screening. Descriptive statistical analyses were performed with STATA 14.2 (StataLP, Corp, TX, USA). To be consistent with the Cochrane review authors, we used RevMan 5.3 [24] and the same model and effect estimates for any meta-analysis recalculations.

## Results

We first summarize the results of the nine rapid reviews across the three case studies and then provide a more in-depth analysis of the findings for each case study. As presented in Table 1 and in the “Methods” section, case study 1 addressed the effectiveness of nivolumab for adult patients with Hodgkin’s lymphoma, case study 2 early versus deferred androgen suppression for advanced prostate cancer, and case study 3 unconditional cash transfers for reducing poverty and vulnerabilities. Table 2 summarizes the characteristics of the Cochrane reviews that we used as reference standards; Additional file 1 also presents the studies that served as each topic’s “starter set” for the abbreviated electronic literature searches of each case study. Table 3 summarizes the abbreviated literature searches and the flow of the literature during screening for each of the nine rapid reviews.

**Table 2:** Characteristics of the Cochrane reviews used as reference standards

	Case study 1: Nivolumab for adult individuals with Hodgkin’s lymphoma [16]	Case study 2: Early versus deferred androgen suppression for treating advanced prostate cancer [18]	Case study 3: Unconditional cash transfers for reducing poverty and vulnerabilities [17]
**Search date**	October 2017 to May 2018	January 23, 2018	May 2, 2017
**Abstracts screened**	675	19,380	30,453
**Excluded abstracts**	626	19,253	30,270
**Full texts screened**	49	127	183
**Excluded full texts**	23	74	115
**Publications included (PubMed indexed)**	26 (4)	53 (34)	68 (11)
**Studies included (PubMed indexed)**	3 (3)	10 (10)	21 (7)
**Studies included in meta-analyses**	0	10	11
**Studies included in the rapid review “starter set”**	2	3	2

**Table 3:** Characteristics of the rapid reviews

	Case study 1: Nivolumab for adult individuals with Hodgkin’s lymphoma			Case study 2: Early versus deferred androgen suppression for treating advanced prostate cancer			Case study 3: Unconditional cash transfers for reducing poverty and vulnerabilities		
**Reviewer**	1	2	3	1	2	3	1	2	3
**Search date**	May 24, 2017 December 08, 2017 (update search)			July 19, 2017			July 10, 2017		
**Publications identified from abbreviated electronic searches**	98			195			385		
**Additional publications identified from searches of reference lists**	1	0	2	2	1	9	49	2	9
**Abstracts screened**	99	98	100	197	196	204	434	387	394
**Excluded abstracts**	94	84	88	182	183	190	337	342	331
**Full texts screened**	5	14	12	15	13	14	97	45	63
**Excluded full texts**	2	11	7	4	5	4	54	22	37
**Publications included**	3	3	4	7	6	7	15	9	12
**Studies included**	3	3	3	6	4	5	10	8	9
**Studies included and identified through reference list checking**	0	0	0	0	0	0	4	3	3
**Studies identified through abbreviated electronic searches but missed by single-reviewer screening**	0	0	0	3	5	4	1	2	1
**Studies included in meta-analyses**	0	0	0	6	4	5	6	4	6

### Changes in conclusions

Our primary question of interest was whether the Cochrane authors would have drawn the same or different conclusions had they relied on a rapid review instead of the Cochrane review. Overall, the conclusions would have been the same as in the Cochrane reports for seven of the nine rapid reviews. For the two oncological topics (case studies 1 and 2), the three rapid reviews for each topic rendered the same conclusions as the respective Cochrane reviews, although the authors would have had less certainty about their conclusion in case study 2. For case study 3, the public health topic, only one of three rapid reviews led to the same conclusion as the Cochrane review. The other two rapid reviews did not contain enough information for the authors to draw conclusions anymore (see Table A2 in Additional file 2).

### Sensitivity to identify relevant studies

For each topic, we assessed the sensitivity of the abbreviated electronic literature search to identify studies that the Cochrane review had included. In addition, we were interested in the combined sensitivity of the abbreviated electronic literature searches, the review of the relevant reference lists, and the single-reviewer screening of the abstracts and full texts.

**Fig. 2:**
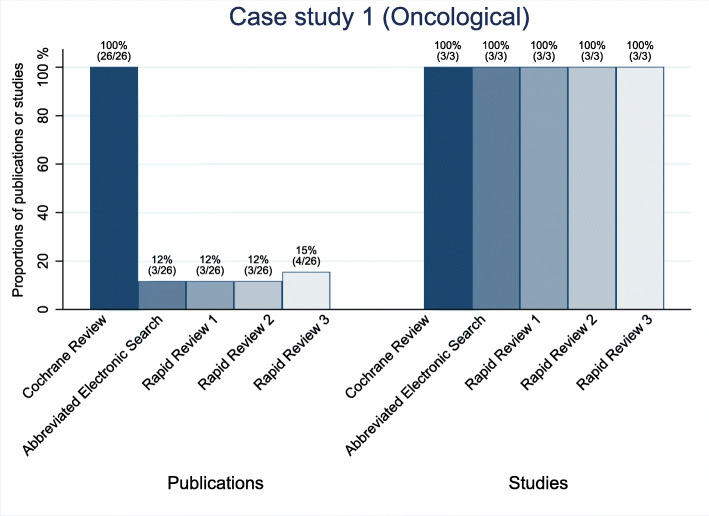
Proportion of the identified publications and studies for case study 1 (oncological)

**Fig. 3:**
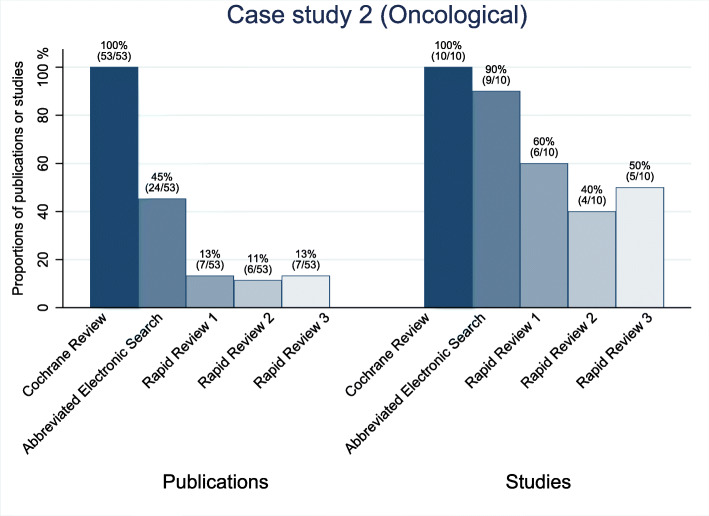
Proportion of the identified publications and studies for case study 2 (oncological)

**Fig. 4:**
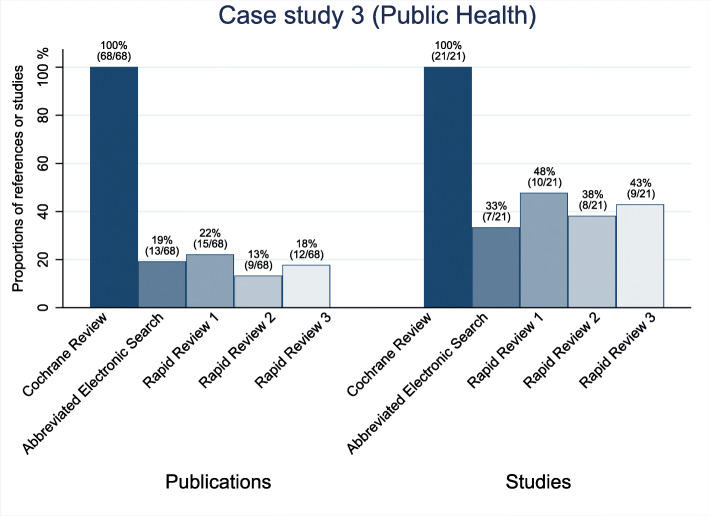
Proportion of the identified publications and studies for case study 3 (public health)

The sensitivity of the abbreviated electronic searches for the two oncological topics was high. They detected 100% (3 of 3) of the included studies for case study 1 and 90% (9 of 10) for case study 2 (see Figs. 2 and 3). For the public health topic (case study 3), the sensitivity of the abbreviated electronic search was only 33% (7 of 21; see Fig. 4). The underlying assumption for these numbers is that the identification of at least one of sometimes several publications of the same study can be equated with the identification of the study. For case study 3, all the studies that the searches did not identify were either gray literature or from journals not indexed in PubMed.

Searching the reference lists did not identify a single missed study in any of the three rapid reviews for case study 2. For case study 3, the reference list searches identified 3 to 4 studies that had been missed by the abbreviated electronic literature searches (see Table 3). In case studies 2 and 3, the single-reviewer screening missed several relevant studies that the abbreviated electronic literature searches had identified. Across these six rapid reviews, the single-reviewer screening missed a median of 31% (range 14 to 56%) of the relevant studies. When we combined the abbreviated electronic searches with the reference list searches and single-reviewer screening, the sensitivity remained at 100% (3 of 3) for case study 1. For case study 2, the single-reviewer screening missed several relevant studies and led to a decrease in the sensitivities for all three rapid reviews. For case study 2, the three rapid reviews identified 40% (4 of 10), 50% (5 of 10), and 60% (6 of 10), respectively; for case study 3, the respective numbers were 38% (8 of 21), 43% (9 of 21), and 48% (10 of 21) (see Figs. 2, 3, 4; Table A1 in Additional file 2).

In the following sections, we summarize each case study in more detail.

### Case study 1

The objective of the Cochrane review for case study 1 was to assess the benefits and harms of nivolumab in adults with Hodgkin’s lymphoma. This Cochrane review [16] included three prospective uncontrolled studies published in 26 publications (see Table 2). Cochrane review authors summarized the results of the three included trials in the main summary of findings’ table narratively. The abbreviated electronic literature searches identified all three studies but only 3 of the 26 publications. The references list searches detected one additional eligible publication for the third rapid review that had been missed by the abbreviated electronic literature searches. Across the three rapid reviews, the single-reviewer screening missed none of the relevant studies identified by the abbreviated electronic literature searches (see Table 3). Based on the abbreviated electronic literature and reference list searches, the rapid reviewers had to screen only small proportions of the abstracts that the investigators screened for the Cochrane review (15% [99 of 675], 15% [98 of 675], and 15% [100 of 675]). The rapid reviewers spent a median of 2.1 h checking the reference lists and the literature screening (abstracts and full texts).

As presented above, the abbreviated electronic literature searches alone and all three rapid reviews identified 100% (3 of 3) of the eligible studies. Consequently, the Cochrane reviewers’ conclusions would not have changed (see Table A2 in Additional file 2). The approach, however, detected only 12% (3 of 26) to 15% (4 of 26) of the relevant publications about these studies. Figure 2 depicts the studies and number of respective publications that each of the three rapid reviews identified for case study 1 (see also Table A1 in Additional file 2).

### Case study 2

The Cochrane review for case study 2 focused on early versus deferred androgen suppression for treating advanced prostate cancer [18]. It identified 10 eligible RCTs published in 53 manuscripts (see Table 2). Cochrane authors included all studies in meta-analyses and presented results in the main summary of findings table. Similar to case study 1, the rapid reviewers had to screen only small proportions of the abstracts screened for the Cochrane review (1% [196 of 19,380], 1% [197 of 19,380], and 1% [204 of 19,380], respectively). The references list searches detected none additional eligible publication that had been missed by the abbreviated electronic literature searches. The reviewers spent a median of 6.5 h screening the literature and scanning the reference lists.

Despite the low number of records that the abbreviated electronic search retrieved (24 of 53), 90% of the relevant studies (9 of 10) were included in the abbreviated electronic search. In case study 2, the sensitivities substantially decreased during the single-reviewer screening. The screeners falsely excluded 3 to 5 relevant studies during the abstract screening.

The sensitivities of the combination of the abbreviated literature searches and single-reviewer screening were 40% (4 of 10), 50% (5 of 10), and 60% (6 of 10), respectively. Of the 53 manuscripts published on the 10 studies, the approach identified between 11% (6 of 53) and 13% (7 of 53). Nevertheless, when asked to base their conclusions on the evidence base of each of the three rapid reviews, the Cochrane review authors would have still drawn the same conclusions, albeit with less certainty (see Table A2 in Additional file 2). Figure 3 depicts the studies and number of respective publications that each of the three rapid reviews identified for case study 2 (see also Table A1 in Additional file 2).

### Case study 3

For case study 3, the Cochrane review [17] focused on unconditional cash transfers to reduce poverty and vulnerabilities and included 21 studies (16 cluster RCTs, 4 controlled before–after studies, and 1 cohort study) summarized in 68 publications (see Table 2). Overall Cochrane review authors included 11 studies in meta-analyses. In the main summary of findings’ tables, they presented results of meta-analyses based on 10 studies and narrative description if results have not been pooled for a certain outcome. Similar to the other case studies, the percentage of the abstracts screened by the rapid reviewers compared to the Cochrane reviewers was low (1% [387 of 30,453], 1% [394 of 30,453], and 1% [434 of 30,453]). The reviewers spent a median of 22.6 h on the screening and searching the reference lists.

The proportion of relevant studies that the abbreviated electronic searches identified (33% [7 of 21], see Fig. 4) was substantially lower for this topic than for the oncological topics. Searching the reference lists added 3 to 4 studies (2 to 7 publications) that had been missed by the abbreviated electronic literature searches across the three rapid reviews (see Table 3).

During the single-reviewer screening, the investigators falsely excluded 1 to 2 studies across the rapid reviews. The sensitivity of the combination of the abbreviated literature search and single-reviewer literature screening on the study level was 38% (8 of 21), 43% (9 of 21), and 48% (10 of 21), respectively. Of the 68 manuscripts published on the 21 studies, the approach identified between 13% (9 of 68) and 22% (15 of 68). The Cochrane review authors would have drawn the same conclusions but with less certainty based on the evidence identified for one of the rapid reviews; however, they would have been unable to draw a conclusion at all had they relied on the evidence identified by the two other rapid reviews (see Table A2 in Additional file 2). Figure 4 depicts the studies and number of respective publications each of the three rapid reviews identified (see also Table A1 in Additional file 2).

## Discussion

To the best of our knowledge, this is the first study that provides data on the validity of a rapid review approach that combines substantially abbreviated literature searches and single-reviewer literature screening. Overall, the results showed that this approach missed between 0% and 67% of the relevant studies and between 78% and 89% of the publications that reported on these studies. The extent of the studies and publications missed strongly depended on the topic. The approach fared better for the oncological topics than for the public health topic. The abbreviated literature searches achieved a substantial reduction in the number of abstracts that needed to be screened (a reduction of 85 to 99%) for the oncological topics. The abbreviated electronic search strategy, however, did not work well for the public health topic. The approach missed 67% of the relevant studies.

The single-reviewer screening was, in general, error-prone. For case studies 2 and 3, the single-reviewer screening missed 14 to 56% of the relevant studies that had been identified by the abbreviated electronic searches. Despite the less than optimal accuracy of this rapid review approach, the Cochrane authors would have drawn the same conclusions for the oncological topics as in the original Cochrane reviews, albeit sometimes with less certainty. For the public health topic, the evidence base from two of the three rapid reviews would have been insufficient to draw conclusions anymore.

The findings of other methods studies assessing the validity of methodological shortcuts for literature searches [10, 13, 25, 26] and single-reviewer screening [12–15] provide, in general, findings consistent with our study. For example, Pham et al. [13] and Bayliss et al. [25] identified 53 to 94% of relevant studies through an abbreviated literature search in one database and ancillary sources (e.g., gray literature search, reference list checking, expert consultations) [13] and a search in MEDLINE only [25]. Two methods studies focusing on the impact on conclusions reported that conclusions changed in 2 to 5% of cases [10, 26]. A recent systematic review on the impact of single-reviewer abstract screening reported that the median proportion of missed studies in four evaluations was 5% (range 0 to 58%) [15].

Our case studies also underpin other studies’ findings [10, 27] that substantially abbreviated searches are more robust for clinical topics than for complex public health questions. Studies of pharmacological interventions are usually published in journals indexed in PubMed and are easy to detect. By contrast, studies related to public health interventions are often not published in journals indexed in PubMed and are sometimes only available in specialized electronic databases or as gray literature [28].

For the two oncological topics (case studies 1 and 2), the abbreviated literature searches yielded substantially fewer abstracts that needed to be screened than the Cochrane reviews (15% and 1% of the Cochrane reviews’ abstracts). For these two topics, the abbreviated electronic searches missed none of the studies for case study 1 and only a single study for case study 2. Assuming that an expert screener can read 100 abstracts per hour, this equals an approximate time savings of 5.7 h per screener for case study 1 and 192 h for case study 2. Across the three case studies, all 15 studies not identified with the abbreviated electronic searches in PubMed were gray literature or published in journals not indexed in PubMed. Therefore, it is likely that an additional abbreviated search for gray literature would identify more studies, also within a short time frame.

Our methods study has several limitations. First, we used a convenience sample of only three topics. Whether our findings are generalizable to other topics remains unclear. Second, our estimates for sensitivity might have been influenced by several necessary methodological decisions. Primarily, we considered the “starter set” (studies that Cochrane authors identified as used for the “similar articles” searches in PubMed) as correctly identified studies. This decision could have led to an overestimation of the sensitivities, although it reflects the methodological approach by Waffenschmidt et al. [11]. In particular for case study 1, the “starter set” contained two out of three eligible studies.

Furthermore, we considered a study identified if at least one publication related to the study was included, although this was not necessarily the publication that contributed data to the quantitative analysis. We had to make this assumption because the Cochrane reviews did not consistently report the exact publications from which they extracted outcome data. This approach might also lead to an overestimation of the sensitivities. In general, for rapid reviews, it is important to identify all relevant studies but not every single publication of a relevant study. Moreover, for case study 2, our searches were conducted earlier than the final searches of the Cochrane review that served as the reference standard. Because of our earlier search dates, we could not identify two studies, which we counted as “missed.” This decision led to an underestimation of the sensitivities for case study 2.

Third, the accuracy of identifying the relevant literature in a systematic review depends on more than literature searches and screening. Systematic reviews, particularly Cochrane reviews, go through extensive peer review, which often identifies studies that the actual systematic review process missed. Therefore, using the final included studies as a reference standard, like in our methods study, sets a very high gold standard. It is conceivable that a subsequent peer review of the rapid reviews would have also identified some of the missed studies.

Fourth, although we randomized the screeners and the topic order to mitigate learning effects and differences in personal expertise, differences in experience and topical knowledge could have influenced the results. The experience among the team of rapid reviewers differed: two reported substantial experience (more than 10 projects), three moderate (3 to 10 projects), and one little experience (fewer than 3 projects). We did not evaluate the reviewers’ familiarity with the topics, which could have also had an impact on the correctness of the screening decisions.

Rapid reviews have become an increasingly used product to support decision-makers. Because of methodological shortcuts, however, the limitations of rapid reviews must be kept in mind. Our findings indicate that their validity varies across topics. For complex public health topics in particular, rapid reviews might not be the right approach to provide a minimum degree of validity. In an international survey of more than 300 decision-makers, the respondents stated that, for rapid reviews to be useful in practice, they expected the same conclusions as in a systematic review in 9 out of 10 products [4]. For public health topics, rapid reviews might not be able to live up to such high expectations. Even for less complex clinical topics, decision-makers must be mindful that any accelerated evidence synthesis product comes with a potential tradeoff in validity. The exact magnitude of validity loss due to methodological shortcuts is difficult to determine and generalize across different topics. Other approaches of accelerating the systematic review process without streamlining methods might be the use of automation tools, experienced systematic reviewers with complementary skills and blocked off time for the duration of the systematic review [29]. However, rapid reviews are usually considered evidence syntheses that apply abbreviated methods and not accelerated systematic reviews [30, 31]. Future large-scale methodological research is necessary to draw firm conclusions regarding the validity of individual methodological shortcuts and the cumulative effects of combining them.

## Conclusions

In conclusion, based on our findings from three case studies, the tested rapid review approach may be feasible for focused clinical questions. For complex public health topics, a combination of abbreviated literature searches and single-reviewer screening does not seem to have the necessary sensitivity to provide an evidence base sufficient for decision-making.

## Supplementary information

**Additional file 1.** Search strategies

**Additional file 2.** Supplementary tables

## Data Availability

The datasets used and analyzed during the current study are available from the corresponding author on reasonable request.

## Abbreviations

RCT: Randomized controlled trial
