# Supplementary material for: Combining abbreviated literature searches with single-reviewer screening: three case studies of rapid reviews
Source: Syst Rev. 2020 Jul 18;9:162. doi: 10.1186/s13643-020-01413-7 (PMC7368980; doi:10.1186/s13643-020-01413-7)
Supplement: Supplementary file 1 — Additional file 1. Search strategies [file 13643_2020_1413_MOESM1_ESM.docx]

## Additional file 1. Search strategies

### **Case study 1**

**PubMed (NLM), Update search December 08, 2017**

| Search | Query | Items found |
| --- | --- | --- |
| [#1](https://www.ncbi.nlm.nih.gov/pubmed) | Search hodgkin lymphoma | [70928](https://www.ncbi.nlm.nih.gov/pubmed/?cmd=HistorySearch&querykey=1) |
| [#2](https://www.ncbi.nlm.nih.gov/pubmed) | Search hodgkins lymphoma | [44585](https://www.ncbi.nlm.nih.gov/pubmed/?cmd=HistorySearch&querykey=2) |
| [#3](https://www.ncbi.nlm.nih.gov/pubmed) | Search nivolumab | [1686](https://www.ncbi.nlm.nih.gov/pubmed/?cmd=HistorySearch&querykey=3) |
| [#4](https://www.ncbi.nlm.nih.gov/pubmed) | Search (#1 or #2) and #3 | [84](https://www.ncbi.nlm.nih.gov/pubmed/?cmd=HistorySearch&querykey=4) |
| [#5](https://www.ncbi.nlm.nih.gov/pubmed) | Search 27451390[uid] | [1](https://www.ncbi.nlm.nih.gov/pubmed/?cmd=HistorySearch&querykey=5) |
| [#6](https://www.ncbi.nlm.nih.gov/pubmed) | Similar articles for PubMed (Select 27451390) | [192](https://www.ncbi.nlm.nih.gov/pubmed/?cmd=HistorySearch&querykey=6) |
| [#7](https://www.ncbi.nlm.nih.gov/pubmed) | Select 20 document(s) | [20](https://www.ncbi.nlm.nih.gov/pubmed/?cmd=HistorySearch&querykey=7) |
| [#8](https://www.ncbi.nlm.nih.gov/pubmed) | Search 25482239[uid] | [1](https://www.ncbi.nlm.nih.gov/pubmed/?cmd=HistorySearch&querykey=8) |
| [#9](https://www.ncbi.nlm.nih.gov/pubmed) | Similar articles for PubMed (Select 25482239) | [141](https://www.ncbi.nlm.nih.gov/pubmed/?cmd=HistorySearch&querykey=9) |
| [#10](https://www.ncbi.nlm.nih.gov/pubmed) | Select 20 document(s) | [20](https://www.ncbi.nlm.nih.gov/pubmed/?cmd=HistorySearch&querykey=10) |
| [#11](https://www.ncbi.nlm.nih.gov/pubmed) | Search #4 or #7 or #10 | [107](https://www.ncbi.nlm.nih.gov/pubmed/?cmd=HistorySearch&querykey=11) |
| [#12](https://www.ncbi.nlm.nih.gov/pubmed) | Search #3 and #11 | [93](https://www.ncbi.nlm.nih.gov/pubmed/?cmd=HistorySearch&querykey=12) |
| [#13](https://www.ncbi.nlm.nih.gov/pubmed) | Search 25656882[UID] OR 27325283[UID] OR 26576863[UID] OR 27622603[UID] OR 26551782[UID] OR 26049755[UID] OR 27881581[UID] OR 25482239[UID] OR 27269741[UID] OR 26929077[UID] OR 27687237[UID] OR 28383639[UID] OR 25639369[UID] OR 26525683[UID] OR 28057180[UID] OR 22658128[UID] OR 26223461[UID] OR 27667773[UID] OR 28359170[UID] OR 28394366[UID] OR 25824455[UID] OR 27899158[UID] OR 27841828[UID] OR 28473905[UID] OR 27991731[UID] OR 28239465[UID] OR 27805626[UID] OR 27932067[UID] OR 28270452[UID] OR 27622997[UID] OR 25856776[UID] OR 27884973[UID] OR 28434018[UID] OR 27942391[UID] OR 27364263[UID] OR 28183681[UID] OR 26560962[UID] OR 28438889[UID] OR 26581237[UID] OR 28488185[UID] OR 28239466[UID] OR 28352133[UID] OR 27117227[UID] OR 28314688[UID] OR 27753058[UID] OR 25384886[UID] OR 25823918[UID] OR 27147112[UID] OR 28267244[UID] OR 27193488[UID] OR 28344662[UID] OR 27739312[UID] OR 27913499[UID] OR 27696192[UID] OR 25986722[UID] OR 28097534[UID] OR 28529947[UID] OR 25704439[UID] OR 25399552[UID] OR 27650634[UID] OR 27733243[UID] OR 28131785[UID] OR 26047524[UID] OR 25482238[UID] OR 27539158[UID] OR 22658127[UID] OR 26577822[UID] OR 26652941[UID] OR 25795410[UID] OR 27269740[UID] OR 23724867[UID] OR 26432723[UID] OR 26828905[UID] OR 27496310[UID] OR 27451390[UID] OR 25999597[UID] | [76](https://www.ncbi.nlm.nih.gov/pubmed/?cmd=HistorySearch&querykey=13) |
| [#14](https://www.ncbi.nlm.nih.gov/pubmed) | Search #12 NOT #13 | [22](https://www.ncbi.nlm.nih.gov/pubmed/?cmd=HistorySearch&querykey=14) |

**PubMed (NLM), May 24, 2017**

| **Search** | **Query** | **Items found** |
| --- | --- | --- |
| [#1](https://www.ncbi.nlm.nih.gov/pubmed) | Search hodgkin lymphoma Sort by: Relevance | [70032](https://www.ncbi.nlm.nih.gov/pubmed/?cmd=HistorySearch&querykey=1) |
| [#2](https://www.ncbi.nlm.nih.gov/pubmed) | Search hodgkins lymphoma Sort by: Relevance | [44048](https://www.ncbi.nlm.nih.gov/pubmed/?cmd=HistorySearch&querykey=2) |
| [#3](https://www.ncbi.nlm.nih.gov/pubmed) | Search nivolumab Sort by: Relevance | [1189](https://www.ncbi.nlm.nih.gov/pubmed/?cmd=HistorySearch&querykey=3) |
| [#4](https://www.ncbi.nlm.nih.gov/pubmed) | Search (#1 or #2) and #3 | [62](https://www.ncbi.nlm.nih.gov/pubmed/?cmd=HistorySearch&querykey=4) |
| [#5](https://www.ncbi.nlm.nih.gov/pubmed) | Search 27451390[uid] | [1](https://www.ncbi.nlm.nih.gov/pubmed/?cmd=HistorySearch&querykey=5) |
| [#6](https://www.ncbi.nlm.nih.gov/pubmed) | Similar articles for PubMed (Select 27451390) | [130](https://www.ncbi.nlm.nih.gov/pubmed/?cmd=HistorySearch&querykey=6) |
| [#8](https://www.ncbi.nlm.nih.gov/pubmed) | Search 25482239[uid] Sort by: Relevance | [1](https://www.ncbi.nlm.nih.gov/pubmed/?cmd=HistorySearch&querykey=8) |
| [#9](https://www.ncbi.nlm.nih.gov/pubmed) | Similar articles for PubMed (Select 25482239) | [219](https://www.ncbi.nlm.nih.gov/pubmed/?cmd=HistorySearch&querykey=9) |
| [#10](https://www.ncbi.nlm.nih.gov/pubmed) | Select 20 document(s) | [20](https://www.ncbi.nlm.nih.gov/pubmed/?cmd=HistorySearch&querykey=10) |
| [#7](https://www.ncbi.nlm.nih.gov/pubmed) | Select 20 document(s) | [20](https://www.ncbi.nlm.nih.gov/pubmed/?cmd=HistorySearch&querykey=7) |
| [#11](https://www.ncbi.nlm.nih.gov/pubmed) | Search #4 or #7 or #10 | [90](https://www.ncbi.nlm.nih.gov/pubmed/?cmd=HistorySearch&querykey=11) |
| [#12](https://www.ncbi.nlm.nih.gov/pubmed) | Search #3 and #11 | [76](https://www.ncbi.nlm.nih.gov/pubmed/?cmd=HistorySearch&querykey=12) |

**Starter Set**

Younes A, Santoro A, Shipp M, Zinzani PL, Timmerman JM, Ansell S, et al. Nivolumab for classical Hodgkin's lymphoma after failure of both autologous stem-cell transplantation and brentuximab vedotin: a multicentre, multicohort, single-arm phase 2 trial. Lancet Oncology 2016;17(9):1283-94.

PMID: 27451390

Ansell SM, Lesokhin AM, Borrello I, Halwani A, Scott EC, Gutierrez M, et al. PD-1 blockade with nivolumab in relapsed or refractory Hodgkin's lymphoma. New England Journal of Medicine 2015;372(4):311-9.

PMID: 25482239

Ansell S, Gutierrez ME, Shipp MA, Gladstone D, Moskowitz A, Borello I, et al. A phase 1 study of nivolumab in combination with ipilimumab for relapsed or refractory hematologic malignancies (CheckMate 039). In: Blood. 2016.

PMID: not indexed in PubMed

### **Case study 2**

**PubMed (NLM), July 19, 2017**

| Search | Query | Items found |
| --- | --- | --- |
| [#1](https://www.ncbi.nlm.nih.gov/pubmed/advanced) | Search prostate cancer | [146777](https://www.ncbi.nlm.nih.gov/pubmed/?cmd=HistorySearch&querykey=1) |
| [#2](https://www.ncbi.nlm.nih.gov/pubmed/advanced) | Search orchiectomy | [16963](https://www.ncbi.nlm.nih.gov/pubmed/?cmd=HistorySearch&querykey=2) |
| [#3](https://www.ncbi.nlm.nih.gov/pubmed/advanced) | Search antiandrogens | [18802](https://www.ncbi.nlm.nih.gov/pubmed/?cmd=HistorySearch&querykey=3) |
| [#4](https://www.ncbi.nlm.nih.gov/pubmed/advanced) | Search immediate OR deferred OR early OR delayed | [1708318](https://www.ncbi.nlm.nih.gov/pubmed/?cmd=HistorySearch&querykey=4) |
| [#5](https://www.ncbi.nlm.nih.gov/pubmed/advanced) | Search #1 AND (#2 OR #3) AND #4 | [1308](https://www.ncbi.nlm.nih.gov/pubmed/?cmd=HistorySearch&querykey=5) |
| [#9](https://www.ncbi.nlm.nih.gov/pubmed/advanced) | Search (Therapy/Narrow[filter]) AND (#5) | [164](https://www.ncbi.nlm.nih.gov/pubmed/?cmd=HistorySearch&querykey=9) |
| [#10](https://www.ncbi.nlm.nih.gov/pubmed/advanced) | Search 16813885[pmid] | [1](https://www.ncbi.nlm.nih.gov/pubmed/?cmd=HistorySearch&querykey=10) |
| [#11](https://www.ncbi.nlm.nih.gov/pubmed/advanced) | Similar articles for PubMed (Select 16813885) | [437](https://www.ncbi.nlm.nih.gov/pubmed/?cmd=HistorySearch&querykey=11) |
| [#12](https://www.ncbi.nlm.nih.gov/pubmed/advanced) | Select 20 document(s) | [20](https://www.ncbi.nlm.nih.gov/pubmed/?cmd=HistorySearch&querykey=12) |
| [#13](https://www.ncbi.nlm.nih.gov/pubmed/advanced) | Search 18823693[pmid] | [1](https://www.ncbi.nlm.nih.gov/pubmed/?cmd=HistorySearch&querykey=13) |
| [#14](https://www.ncbi.nlm.nih.gov/pubmed/advanced) | Similar articles for PubMed (Select 18823693) | [86](https://www.ncbi.nlm.nih.gov/pubmed/?cmd=HistorySearch&querykey=14) |
| [#15](https://www.ncbi.nlm.nih.gov/pubmed/advanced) | Select 20 document(s) | [20](https://www.ncbi.nlm.nih.gov/pubmed/?cmd=HistorySearch&querykey=15) |
| [#16](https://www.ncbi.nlm.nih.gov/pubmed/advanced) | Search 16622261[pmid] | [1](https://www.ncbi.nlm.nih.gov/pubmed/?cmd=HistorySearch&querykey=16) |
| [#17](https://www.ncbi.nlm.nih.gov/pubmed/advanced) | Similar articles for PubMed (Select 16622261) | [161](https://www.ncbi.nlm.nih.gov/pubmed/?cmd=HistorySearch&querykey=17) |
| [#18](https://www.ncbi.nlm.nih.gov/pubmed/advanced) | Select 20 document(s) | [20](https://www.ncbi.nlm.nih.gov/pubmed/?cmd=HistorySearch&querykey=18) |
| [#19](https://www.ncbi.nlm.nih.gov/pubmed/advanced) | Search #12 OR #15 OR #18 | [48](https://www.ncbi.nlm.nih.gov/pubmed/?cmd=HistorySearch&querykey=19) |
| [#20](https://www.ncbi.nlm.nih.gov/pubmed/advanced) | Search #9 OR #19 | [195](https://www.ncbi.nlm.nih.gov/pubmed/?cmd=HistorySearch&querykey=20) |

**Starter Set**

Schröder F.H.;K, K-H.; Fossa S.D.; Hoekstra W.; Karthaus P.P.; De Prijck L.; Collette L. Early Versus Delayed Endocrine Treatment of T2-T3 pN1-3 M0 Prostate Cancer Without Local Treatment of the Primary Tumour: Final Results of European Organisation for the Research and Treatment of Cancer Protocol 30846 After 13 Years of Follow-up (A Randomised Controlled Trial). European Urology 2009;55(1):14-22.

PMID: 18823693

Studer U.E.; Whelan P.; Albrecht W.; Casselman J.; de Reijke T.; Hauri D.; Loidl W.; Isorna S.; Sundaram S.K.; Debois M.; Collette L. Immediate or deferred androgen deprivation for patients with prostate cancer not suitable for local treatment with curative intent: European Organisation for Research and Treatment of Cancer (EORTC) Trial 30891. Journal of Clinical Oncology 2006;24(12):1868-76.

PMID: 16622261

Granfors, T.; Modig, H.; Damber, J. E.; Tomic, R.. Long-Term Followup of a Randomized Study of Locally Advanced Prostate Cancer Treated With Combined Orchiectomy and External Radiotherapy Versus Radiotherapy Alone. Journal of Urology 2006;176(2):544-7.

PMID: 16813885

### **Case study 3**

**PubMed (NLM), July 10, 2017**

| Search | Query | Items found |
| --- | --- | --- |
| [#1](https://www.ncbi.nlm.nih.gov/pubmed/advanced) | Search cash transfer Sort by: Relevance | [363](https://www.ncbi.nlm.nih.gov/pubmed/?cmd=HistorySearch&querykey=1) |
| [#2](https://www.ncbi.nlm.nih.gov/pubmed/advanced) | Search 22341825[uid] Sort by: Relevance | [1](https://www.ncbi.nlm.nih.gov/pubmed/?cmd=HistorySearch&querykey=2) |
| [#3](https://www.ncbi.nlm.nih.gov/pubmed/advanced) | Similar articles for PubMed (Select 22341825) | [219](https://www.ncbi.nlm.nih.gov/pubmed/?cmd=HistorySearch&querykey=3) |
| [#4](https://www.ncbi.nlm.nih.gov/pubmed/advanced) | Select 20 document(s) | [20](https://www.ncbi.nlm.nih.gov/pubmed/?cmd=HistorySearch&querykey=4) |
| [#5](https://www.ncbi.nlm.nih.gov/pubmed/advanced) | Search 23453283[uid] Sort by: Relevance | [1](https://www.ncbi.nlm.nih.gov/pubmed/?cmd=HistorySearch&querykey=5) |
| [#6](https://www.ncbi.nlm.nih.gov/pubmed/advanced) | Similar articles for PubMed (Select 23453283) | [161](https://www.ncbi.nlm.nih.gov/pubmed/?cmd=HistorySearch&querykey=6) |
| [#7](https://www.ncbi.nlm.nih.gov/pubmed/advanced) | Select 20 document(s) | [20](https://www.ncbi.nlm.nih.gov/pubmed/?cmd=HistorySearch&querykey=7) |
| [#8](https://www.ncbi.nlm.nih.gov/pubmed/advanced) | Search #1 or #4 or #7 | [385](https://www.ncbi.nlm.nih.gov/pubmed/?cmd=HistorySearch&querykey=8) |

**Starter Set**

Baird SJ, Garfein RS, McIntosh CT, Ozler B. Effect of a cash transfer programme for schooling on prevalence of HIV and herpes simplex type 2 in Malawi: a cluster randomised trial. Lancet 2012;379(9823):1320-9.

PMID: 22341825

Robertson L, Mushati P, Eaton JW, Dumba L, Mavise G, Makoni J, et al. Effects of unconditional and conditional cash transfers on child health and development in Zimbabwe: a cluster-randomised trial. Lancet 2013;381(9874):1283-92.

PMID: 23453283
