# Supplementary material for: Combining abbreviated literature searches with single-reviewer screening: three case studies of rapid reviews
Source: Syst Rev. 2020 Jul 18;9:162. doi: 10.1186/s13643-020-01413-7 (PMC7368980; doi:10.1186/s13643-020-01413-7)
Supplement: Supplementary file 2 — Additional file 2. Supplementary tables [file 13643_2020_1413_MOESM2_ESM.docx]

## Additional file 2. Supplementary tables

**Table A1.** Summary of the case studies

|  | **Case study 1: Nivolumab for adult individuals with Hodgkin’s lymphoma** | | | **Case study 2: Early versus deferred androgen suppression for treating advanced prostate cancer** | | | **Case study 3 Unconditional cash transfers for reducing poverty and vulnerabilities** | | |
| --- | --- | --- | --- | --- | --- | --- | --- | --- | --- |
| **Reviewer** | 1 | 2 | 3 | 1 | 2 | 3 | 1 | 2 | 3 |
| **Screened abstracts in the RR compared to the CR (%)** | 14.7 | 14.5 | 14.8 | 1.0 | 1.0 | 1.1 | 1.4 | 1.3 | 1.3 |
| **Sensitivity for studies in the abbreviated electronic search compared to the CR (%)** | 100.0 | | | 90.0 | | | 33.3 | | |
| **Sensitivity for studies in the RR compared to the CR (%)** | 100.0 | 100.0 | 100.0 | 60.0 | 40.0 | 50.0 | 47.6 | 38.1 | 42.9 |
| **Studies missed by the single-reviewer screening that the abbreviated literature search had identified (%)** | - | - | - | 33.3 (3/9) | 55.6 (5/9) | 44.4 (4/9) | 14.3 (1/7) | 28.6 (2/7) | 14.3 (1/7) |
| **Total reviewer hours (h)** | 4.0 | 1.6 | 2.1 | 6.5 | 6.5 | 4.5 | 30.2 | 15.0 | NR |
| **Median hours (h) (interquartile range)** | 2.1 (1.8–3.0) | | | 6.5 (5.5–6.5) | | | 22.6 (18.8–26.4) | | |

**Abbreviations:** CR = Cochrane review, h = hours, NR = not reported, RR = rapid review approach, % = percentage

**Table A2.** Results from the survey asking review authors whether their conclusion would change

| **Review topic** | **Rapid**  **Review***  **1** | **Rapid**  **Review***  **2** | **Rapid**  **Review***  **3** | **Proportion of concordant conclusions** |
| --- | --- | --- | --- | --- |
| **Case study 1: Nivolumab for adults with Hodgkin’s lymphoma (Pharmacologic)** | The conclusion does not change. | The conclusion does not change. | The conclusion does not change. | 3/3 (100%) |
| **Case study 2:**  **Early versus deferred androgen suppression for treating advanced prostate cancer**  **(Pharmacologic)** | I would draw the same conclusion but with less certainty. | I would draw the same conclusion but with less certainty. | I would draw the same conclusion but with less certainty. | 3/3 (100%) |
| **Case study 3: Unconditional cash transfers for reducing poverty and vulnerabilities**  **(Public Health)** | I would draw the same conclusion but with less certainty. | I can no longer draw a conclusion. | I can no longer draw a conclusion. | 1/3 (33%) |

*Abbreviated search and single-reviewer literature review
